# Laboratory medicine and sports: where are we now?

**DOI:** 10.11613/BM.2024.030501

**Published:** 2024-08-05

**Authors:** Lovorka Đerek, Vanja Radišić Biljak, Sanja Marević, Brankica Šimac, Marko Žarak, Antonija Perović, Domagoj Marijančević, Robert Buljubašić, Luka Matanović, Maja Cigrovski Berković

**Affiliations:** 1 Clinical Department for Laboratory Diagnostics, University Hospital Dubrava, Zagreb, Croatia; 2 School of Medicine, Catholic University of Croatia, Zagreb, Croatia; 3 Department of Medical Laboratory Diagnostics, University Hospital Sveti Duh, Zagreb, Croatia; 4 Faculty of Kinesiology, University of Zagreb, Zagreb, Croatia; 5 Faculty of Pharmacy and Biochemistry, University of Zagreb, Zagreb, Croatia; 6 Medical Biochemistry Laboratory, Health Care Institution Glavić, Dubrovnik, Croatia; 7 Faculty of nursing and clinical nursing, University of Dubrovnik, Dubrovnik, Croatia; 8 Department of Clinical Chemistry, Sestre milosrdnice University Hospital Center, Zagreb, Croatia; 9 Department for Orthopedics and Traumatology, Clinic for Surgery, University Hospital Dubrava, Zagreb, Croatia

**Keywords:** sports, medical laboratory science, endocrinology, orthopaedics, biological variation

## Abstract

Laboratory medicine in sport and exercise has significantly developed during the last decades with the awareness that physical activity contributes to improved health status, and is present in monitoring both professional and recreational athletes. Training and competitions can modify concentrations of a variety of laboratory parameters, so the accurate laboratory data interpretation includes controlled and known preanalytical and analytical variables to prevent misleading interpretations. The paper represents a comprehensive summary of the lectures presented during the 35^th^ Annual Symposium of the Croatian Society of Medical Biochemistry and Laboratory Medicine. It describes management of frequent sport injuries and sums up current knowledge of selected areas in laboratory medicine and sports including biological variation, changes in biochemical parameters and glycemic status. Additionally, the paper polemicizes sex hormone disorders in sports, encourages and comments research in recreational sports and laboratory medicine. In order to give the wider view, the connection of legal training protocols as well as monitoring prohibited substances in training is also considered through the eyes of laboratory medicine.

## Graphical abstract

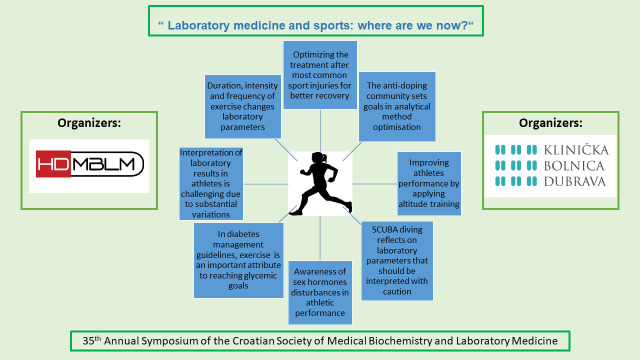

## Highlights

• Training can modify concentrations of a variety of laboratory parameters

## Introduction

Laboratory medicine in sport and exercise is not an old branch of laboratory science, but it has developed during the last decades with the awareness that physical activity contributes to improved health status (*1*, *2*). In that sense, laboratory medicine is present in monitoring both professional and recreational athletes. Knowing that sport can provide benefits in health as well as in disease, laboratory medicine in sports is considered to be a preventive science. Evaluation of the condition of an athlete includes monitoring of workloads and recovery period using various laboratory tests all to achieve top performance (*3*). That way it is possible to screen for injuries or medical conditions that can endanger the athlete as well as to optimize the training and recovery process (*2*, *3*). Training and competitions can modify concentrations of a variety of laboratory parameters, so the accurate laboratory data interpretation includes controlled and known preanalytical and analytical variables to prevent misleading interpretations (*4*). Sports physicians collect extensive amounts of clinical information, which, when combined with biochemical data, can improve understanding of sports physiology and pathology. Nevertheless, caution is necessary in their interpretation because the reference ranges differ between sedentary populations, recreational athletes, and especially elite athletes. Clinical interpretation of laboratory abnormalities in sport often does not reflect the onset of pathology, but could reflect a decrease of performances. That way it could lead to better management of overtraining and overreaching and defining athlete profile that all adds to preventive science description (*5*).

This paper gives an overview of the lectures presented at the 35th annual symposium ˝Laboratory medicine and sport: where are we now?“ organized by University Hospital Dubrava and under the auspices of Croatian Society of Medical Biochemistry and Laboratory Medicine (CSMBLM).

## Biological variability in athletes

Beginning with the pioneer work of Ricos *et al.* and the first integrated table of analytes and derived laboratory performance specifications, freely available at the Westgard quality control web page, through 2014 European Federation of Clinical Chemistry and Laboratory Medicine (EFLM) 1st Strategic Conference for the defining analytical performance specifications, where analytical performance specifications were defined, among other models, by the model based on components of biological variation (BV) of the measurand, to the new EFLM updated biological variation database based on the strict meta-analysis of the published studies, biological variability has become an integrated part of laboratory medicine (*6*-*8*). The BV data are reference data that have many applications in laboratory medicine. The biological variability data describe the variability of clinically important measurands around homeostatic set points within subjects (intra-individual coefficient of variation - CVI) and between subjects (inter-individual CV), which enables the interpretation of laboratory results in clinical settings through reference change value (RCV). In a steady state setting, the concentration of most measurands is characterized by random variation around a homeostatic set point, whereas the concentration of some measurands is also influenced by different life phases or predictable cyclic variation (*9*). One of the newest possible applications of BV data is establishing individual reference intervals (*9*). However, it is worth emphasizing that the BV literature data is available mostly from studies performed on healthy non-athlete individuals. It is well known that intensive physical activity causes significant functional and metabolic changes and adaptations in the athlete’s organism. These adaptation changes encompass cells, tissues, organs, as well as total body physiological changes (*10*). The observed physiological changes result in scarcely described altered biochemical and hematological parameters. However, the newest initiative in anti-doping testing relies exclusively on observing the long-term physiological changes in the hematological and steroidal biochemical parameters in the blood of the athlete, the so-called Athlete Biological Passport (APB) (*11*). The APB comprises repeated measurements of hematological and steroidal markers which enables to establishment of the athlete’s laboratory results baseline. The APB longitudinally follows the athlete’s collected data and the possible observed differences are defined by BV. As a high level of physical exercise most probably influences BV, the studies performed on healthy non-athlete volunteers cannot be simply transferred to athletes (*12*). Recently, some BV studies performed on athletes were published. Diaz-Garzon *et al.* observed higher CVI estimates for routine laboratory biochemistry measurands in athletes than what has been reported for the general population (*13*). The observed differences may be related to physiological stress over time caused by the continuous practice of exercise. However, in a similar study performed by the same group of authors, CVI estimates of most hematological parameters in a group of recreational endurance athletes were similar to the general population and were not influenced by exercise or athletes’ state of health (*14*). It is evident from these conflicting results that there is substantial variability even among athletes, and future studies regarding BV data in this particular group of males and females are needed.

## Effects of physical activity on biochemical tests

A sedentary life style can raise risks for various diseases, such as diabetes mellitus (DM), cardiovascular diseases (CVD), hypertension, but also cancer, so physical exercise is of utmost importance for the overall health status. Biochemical and hematological tests are prone to changes in blood of individuals, depending on the duration, intensity and frequency of physical exercises. These changes, which are not considered as preanalytical errors, may lead to benefits, such as reduced risk factors as lower lipid blood profile, but also to pathophysiological changes, such as cell injuries. Interpretation of these changes in the context of physically active subjects is crucial. Although, the majority of literature is investigating these parameters in professional athletes, there are evidences about many beneficial effects in average individuals but also in people with some morbidities (*15*-*17*).

Although, physical activity is an important preanalytical factor in biochemical tests, according to good laboratory practice (GLP) individuals should *prior* to phlebotomy, avoid physical activity at least 48 h (*15*). However, this GLP can’t always be respected when a patient is admitted to the emergency department.

The influence of physical exercise on a variety of common biochemical parameters are well known. First, there are changes of plasma volume which depend on the type of training exercises, so when interpreting laboratory tests, one should always take into consideration the extent of the possible hemoconcentration and/or hemodilution (*15*, *16*). Elevation of serum enzymes from the skeletal muscles like creatine kinase, aspartate aminotransferase (AST) and lactate dehydrogenase, depends on the duration and intensity of exercise, with highest values in untrained individuals (*16*).

According to literature, physical exercise in healthy women and men are related to higher HDL, lower LDL and triglycerides values, which contributes to lower risk for CVD (*18*-*20*). However, lipoprotein(a) (Lp(a)) and apolipoprotein B (apoB) remain unchanged (*19*). Additionally, lower concentrations of glucose, glycated haemoglobin (HbA1c), C-reactive protein (CRP), estimated glomerular filtration rate but higher concentrations of creatinine, iron, plasma calcium after aerobic or strength exercise are present (*18*). However, most of these parameters remain in the reference intervals, but there should be care regarding interpretation of these parameters, especially creatinine concentrations (*18*). In context of the type and frequency of exercise or gender, various influences are observed regarding concentrations of total cholesterol, AST, gamma-glutamyl transferase, alkaline phosphatase, uric acid, total bilirubin, and total iron binding capacity (*18*). The most frequent cause of anemia is iron deficiency, especially in female endurance athletes (*16*).

In patients with DM type 2, high intensity interval training resulted in whole body mass decrease, but also in lower concentrations of fasting and postprandial glucose and HbA1c (*21*).

## Diagnosing and preventing hypoglycemia during and after exercise

Exercise is advocated in diabetes management guidelines as an important attribute to reaching glycemic goals and avoiding vascular complications in both people with type 2 and type 1 diabetes. On the other hand, exercise-related hypoglycemia is a major concern of people with diabetes; primarily those on insulin treatment (*22*). Therefore, balancing between regular exercise (and sports) participation and attainment of normoglycemia is of utmost importance, and modern technology in terms of improved insulins, means of their delivery, and glucose monitoring systems, is an important accomplice on this path (*23*). In addition, choosing the right exercise type and intensity and timing of the exercise during the day, can all influence exercising safety, *i.e.,* minimize the risk of glycemic excursions into hyper- or hypoglycemic direction. Although the use of technology, especially continuous glucose monitoring (CGM) and intermittently scanned CGM made around-exercise glucose management safer and easier, there is still a need to extrapolate from the huge amount of data gained from technology to implement it in the user-friendly real-life setting and accommodate quick-decision making which is especially important during exercise and sport (*24*). The possible limitations related to new glucose monitoring systems must be taken into account. Mentioned primarily relates to acknowledging the lag time, which might be crucial during the exercise, where physiological factors such as body temperature and acidity related to working muscle and blood flow alterations can impact the sensor-measured accuracy of interstitial glucose concentrations. In addition, there are unanswered questions related to the positioning of the sensor in the context of different sports *i.e.,* should for example cyclist wear the sensor on the leg instead of the forearm to increase the accuracy of glucose measurements? Yet another important aspect of technology use in sports is its implementation in sports nutrition and understanding of how interstitial glucose influences muscle fuelling in the setting of training or competition. Therefore, CGM use is an important tool to help prevent hypoglycemia during or after exercise, understand how different foods influence insulin sensitivity and therefore optimize pre-exercise/competition nutrition (*25*-*27*). Current guidelines attempted to set safe glucose targets around the exercise for people with type 1 diabetes concerning the intensity of exercise they participate in and based on their risk of hypoglycemia, incorporating the glucose trends gathered from sensor glucose measurements. Generally, the safe glucose ranges for starting the exercise and during the exercise is between 7 and 12 mmol/L. At the same time, values below or above this target might prompt carbohydrate intake and/or insulin dose reduction or postponement of exercise and insulin bolus and hydration, respectively (*22*, *24*). But it is important to keep in mind that each person is an individual and therefore guidelines are just provisory, while CGM will give individual insights that must be used as a learning tool to offer true precision care.

## Sex hormone disorders - the other side of sports

Androgens have the potential to enhance athletic performance by influencing the structure of muscle tissue, bone mass, erythropoietin (EPO) effects, the immune system and behavioural patterns (*28*, *29*). Experimental findings indicate that testosterone increases skeletal muscle myostatin concentration, mitochondrial biogenesis, myoglobin expression and insulin-like growth factor I muscle content, potentially increasing skeletal muscle activity (*30*). Androgens promote bone growth, both directly and indirectly, through the local aromatization of estrogens (*31*). Increased bone mass and strength may be advantageous for sports involving explosive motions like throwing and jumping. Additionally, testosterone increases circulating hemoglobin concentrations and promotes the production of new erythrocytes, presumably by inducing lower hepcidin and higher EPO secretion (*32*, *33*). Furthermore, there was a positive correlation observed between the athletes’ performance and serum concentrations of dihydrotestosterone (DHT) and dehydroepiandrosterone (DHEA) (*34*). This finding is important since growing evidence suggests that DHEA is the major precursor of bioactive androgens in women, intracellularly transformed into testosterone and DHT, which bind to the androgen receptor (*35*). All of these results show that lean mass and physical performance have a positive correlation with endogenous androgen concentrations, regardless of whether they are beyond the reference intervals. In comparison to other female athletes with the same body mass index (BMI), endurance athletes with polycystic ovary syndrome (PCOS) perform significantly better in the multi-stage fitness test to determine a person’s aerobic capacity and demonstrate higher maximal oxygen uptake (VO2 max) during the treadmill fatigue test (*36*). Regardless of body composition, the PCOS women had superior muscle strength (bench press, leg extension, and handgrip strength). In the PCOS group, there was a positive association between elevated muscle strength and serum testosterone concentrations (*37*). Additionally, Olympic athletes had higher rates of PCOS and polycystic ovaries (*38*). These findings suggest that moderate forms of hyperandrogenism, especially PCOS, may enhance physical performance and affect women’s decision to participate in competitive sports. The concept of reverse causality is unsupported. Elite female athletes are more likely to have XY differences/disorders of sex development, which implies that these disorders may enhance physical performance. According to predictions, having testosterone concentrations in the “male” range gives female athletes an ergogenic advantage of more than 9% (*39*).

Hackney and colleagues described the rate of testosterone reduction required to identify an athlete as having the “Exercise Hypogonadal Male Condition” (*40*, *41*). These researchers proposed this differentiation as a relative form of functional hypogonadotropic hypogonadism required sustained testosterone concentrations at least 25% to 50% lower than expected for their age, representing a potential adaptive response in the hypothalamic-pituitary-gonadal (HPG) axis, from chronic, long-term exercise exposure. Evidence indicates that in males with Overtraining Syndrome and/or Triad/“Reduced Energy Deficiency in Sports” condition, a decrease in testosterone caused by exercise-induced relative hypogonadism is harmful. These individuals are unable to compete at their highest level or full potential due to deprived health and physical performance. Such individuals are suffering from a well-known endocrine dysfunction. However, it is crucial to remember that low testosterone-hypogonadism can occur in athletes as a result of additional circumstances, such as traumatic brain injuries incidents or anabolic androgenic steroids usage (*42*, *43*).

## Recreational diving and laboratory medicine - can the depths of the sea change the results of laboratory tests?

Croatia is a Mediterranean country whose tourism activity is for its most part directly connected to the coast of the Adriatic Sea. According to the data of the Croatian Diving Association, which is a member of the World Diving Confederation (CMAS), 150 diving clubs and 150 diving centres are currently registered in Croatia (*44*). Since recreational SCUBA (self-contained underwater breathing apparatus) diving has become a very popular sport in the last 20 years, with millions of recreational divers worldwide and thousands in Croatia every year, there is a need to understand how it affects the physiology of the human body and whether the sea depths can affect the results of laboratory tests in healthy individuals who practice this sport recreationally. This information is especially important for laboratory professionals who work in laboratories located in the coastal region of Croatia.

SCUBA diving is a special form of physical activity that, due to changed environmental conditions (exposure to the hyperbaric conditions, elevated breathing pressure, effect of immersion, exposure to cold temperature) together with increased physical load, triggers stress response of the organism (*45*). According to their purpose, modern forms of diving can be divided into amateur (sports/recreational) and professional (technical) diving, of which more common is recreational diving. European (EN 14153-2) and international standards (ISO 24801-2) together with the CMAS documentation define recreational SCUBA diving as a form of diving limited to depths up to 40 meters using only compressed air or nitrox (a gas mixture of oxygen and nitrogen, where the proportion of oxygen does not exceed 40%) with direct, vertical access to the surface and gradual surfacing without decompression stop (*46*).

Numerous Croatian studies on Croatian divers have shown that all forms of diving can affect the results of laboratory tests that indicate activation, adaptation, or impairment (transient or permanent) of specific organ or organic system, depending on depth, duration and repetition (acute or chronic/repetitive effect). Those changes can be, not only statistically, but also clinically significant in healthy individual as compared to biological variation of specific parameters (RCV). Up to date, we know that SCUBA diving can cause changes in routine hematological parameters and erythropoiesis (*47*, *48*). It can also change oxidant/antioxidant status of the whole organism (*49*). Many studies have noticed transient impairment and adaptation of cardiac, muscular, and immune systems (*50*-*52*). Furthermore, it has recently been discovered that recreative SCUBA diving can also impact neurohormonal response and myokines-mediated communication between muscles and the brain (*53*). Finally, there are many other routine laboratory results that do not change after diving. The important goal of the further studies on Croatian population of divers should be inclusion of more participants, since all the studies have been performed on maximum of 15 to 20 divers.

As a concluding remark, the effects that recreative SCUBA diving induces in numerous organs and organic systems merit the attention and caution of clinicians and laboratory professionals when interpreting the laboratory test results in people who practice the sport. The added value brought by the presented studies is a translational potential in improving the health check strategy for professional divers.

## The oxygen paradox - can intermittent hyperoxia have similar effects as altitude training?

In many sports, altitude or hypoxic training has become a standard training protocol before important competitions. There are several models of hypoxic training, such as live high-train high (LH-TH), live high-train low (LH-TL) and live low-train high (LL-TH), and the purpose of all of them is to stimulate adaptation mechanisms to a hypoxic condition resulting in improved endurance of athletes in a normoxic environment (*54*). Hypoxia provokes a natural and multiple response in the organism, activating biological processes at the cellular level and affecting various systems such as the hematopoietic, metabolic, respiratory, and cardiovascular (*55*). The hypoxic stress inside cells activates a family of transcriptional factors called Hypoxia Inducible Factors (HIFs) (*56*). HIFs modulate the response to hypoxia by prompting the expression of hundreds of genes that are involved in metabolism regulation, erythrocyte production, angiogenesis, cell growth/death, proliferation and differentiation, glycolysis, mitochondrial metabolism, immune and inflammatory response (*57*). For athletes, the key effect of altitude training or hypoxia exposure is the stimulation of EPO production, which accelerates erythropoiesis and oxygen delivery to the cells (*58*).

It has also been shown that oxygen concentration fluctuations, as a consequence of exposure to hyperoxia, can have a similar effect as hypoxia on EPO concentrations (*55*). A phenomenon known as the “oxygen paradox” was first observed and described in healthy subjects after breathing 100% oxygen at normal pressure (exposure to normobaric hypoxia for 2 h) (*59*). Later, an increase in EPO concentration was also observed after saturation dives (long-term exposure to hyperbaric hyperoxia) and recreational SCUBA diving performed once a week for five consecutive weeks (continuous short-term intermittent hyperbaric hyperoxia) (*48*, *60*, *61*). According to the “oxygen paradox” hypothesis, the return to normoxia, after exposure to hyperoxia, is interpreted by cells as hypoxia and gene expression regulated by HIFs is stimulated. It is postulated that these complex cellular signaling mechanisms are associated with changes in the oxidant-antioxidant status during hyperoxia and normoxia resulting in the stabilization of HIFs (*62*).

The EPO response after hyperoxic exposure opens up many areas of interest, not only for the improvement of athletes’ performance. Although the “oxygen paradox” theory provides a promising strategy for increased EPO production, further studies are needed.

## Laboratory testing of athletes to detect doping

The Festina affair was a series of doping scandals in professional cycling that occurred during and after the 1998 Tour de France. This event prompted worldwide sports organisations to take action, leading to the establishment of the international independent World Anti-Doping Agency (WADA) in 1999. WADA’s main task is to develop, promote and coordinate anti-doping rules and measures in all sports and countries in order to ensure doping-free sport (*63*). According to the traditional definition, doping is the use of prohibited drugs or methods with the aim of improving psychophysical abilities. Today, doping is defined as one or more violations of the anti-doping rules laid down in the World Anti-Doping Code adopted by WADA (*64*).

The main document listing substances and methods of doping in sport is the WADA prohibited list, which is updated annually. In 2024, it includes 11 classes of prohibited substances (S0-S9 plus P1) and three categories of prohibited methods (M1-M3) (*65*). Athletes’ samples may only be analyzed in WADA-accredited laboratories, of which there are currently around 30 worldwide (*66*). Before WADA grants accreditation, laboratories must be accredited in accordance with the requirements of ISO/IEC 17025. Laboratories that are not accredited by WADA may be approved by WADA to perform blood sample analyses in support of the hematology module of the ABP. Athlete Biological Passport laboratories may be accredited to either ISO/IEC 17025 or ISO 15189 (*67*).

Laboratories must follow WADA’s International Standard for Laboratories to ensure that they report valid test results and facilitate the harmonisation of analytical testing of samples (*67*). Laboratories are required to perform all prescribed analytical test procedures set out by WADA in specific technical documents, technical letters or laboratory guidelines (*68*).

For most anabolic androgenic steroids and other anabolic agents, as well as for growth hormone, its fragments and releasing factors, β2-agonists, hormone and metabolic modulators and stimulants, the method of choice is gas (GC) or liquid chromatography coupled to mass spectrometry (MS) for both the initial testing procedure and the confirmation procedure in case of an initial adverse analytical finding or an atypical finding (*67*). A more specific example refers to a case of suspicious steroid profile data, when the confirmatory method of choice is GC/combustion/Isotope Ratio MS to determine if significant differences exist between the carbon isotope signatures of target compounds and endogenous reference compounds such as pregnanediol, pregnanetriol, 11β-hydroxy-androsterone and 11-oxo-etiocholanolone (*69*).

Electrophoretic methods, affinity binding assays (*e.g.* immunoassays) and other analytical methods are also routinely used to detect various macromolecules in samples. It is important to note that affinity binding assays must differ in the use of affinity reagents (*e.g.* antibodies) depending on whether they are used for initial or confirmatory testing. They should recognise different epitopes of the macromolecule being analyzed, unless a purification (*e.g.* immunopurification) or separation method (*e.g.* electrophoresis, chromatography) is used *prior* to the application of the affinity binding assay to eliminate potential cross-reactivity (*67*).

The further optimisation and refinement of analytical methods, the expansion of knowledge about the metabolism and disposition of drugs, but also about the possibilities of eliminating interfering factors, are currently the most important goals of laboratory medicine-related research in the anti-doping community (*70*).

## Achilles tendon rupture - operative *versus* nonoperative treatment - does the room for the laboratory medicine appear?

Achilles tendon ruptures (ATR) are among the most common injuries in professional and recreational athletes with estimated annual incidence between 13 and 55 *per* 100,000 (*71*, *72*). Due to the possibility of severe functional limitations of professional athletes, a rational decision about optimal treatment has to be made on evidence-based principles. The summary of the dilemma when choosing between operative and conservative treatment is that the surgical approach yields a lower re-rupture risk but concomitantly associates with an increase in complication risks such as infections or nerve damage (*72*). The surgeon chooses the treatment method individually based on the clinical examination, patient characteristics, and expectations, combined with the ultrasound findings. In the moment of deciding for the type of treatment, laboratory findings do not play a significant role. Nevertheless, they are helpful in perioperative days serving as reliable indicators of efficient and fast recovery by better monitoring of patient fitness status and timely detection of the complication. Nowadays, there is a shifting trend among Orthopedics and Traumatology surgeons with an increased number of studies suggesting that operative treatment of acute ATR is not superior to conservative methods.

A literature review of the current knowledge in treating acute traumatic ATR was performed in order to test the hypothesis that nonoperative treatment is „equivalent“ to operative treatment in terms of re-rupture rate and long-term patient satisfaction. We have searched the PubMed database for the terms „Achilles tendon rupture“ and „operative *vs* nonoperative“ and obtained 27 results. After including the studies published from 2020 until 2024, and excluding inapplicable studies, we selected 5 studies that matched the topic.

Of the 5 studies included in this research, 3 were comparing patient satisfaction, re-rupture rate and complication rate, while 2 studies provided the cost-benefit analysis of operative *vs* nonoperative treatment. The oldest study from this group was published in 2020 which concluded that there was no demonstrable difference in patient-reported outcome, satisfaction, or re-rupture rates at long-term follow-up (*73*). Next study from 2022 suggested that nonoperative treatment of ATR may have up to 6.4% higher relative risk of re-rupture and that previous studies were only comparing the absolute risk of re-rupture (*74*). The newest study from this group was published in 2023 indicated no differences in reoperation rates between operative and nonoperative management of ATR (*75*). Also, operative management was associated with an increased risk of complications and higher initial costs, which dissipated over time. Both cost-benefit analyses were on average in favour of nonoperative treatment (*71*, *75*). Surgical repair is associated with greater costs partially because of the greater utilization of clinic visits, imaging, and physical therapy sessions. Nonoperative treatment is associated with higher prescription costs secondary to a longer duration of opioid use.

Current papers available on this topic prove that nonoperative treatment has similar long-term patient satisfactory results compared to operative management. Furthermore, the cost of nonoperative treatment seems to be less, which is also a factor that should not be ignored. Still, decisions on treatment should be made by the surgeon on an individual basis. Helping tools such as Copenhagen Achilles Rupture Treatment Algorithm could help in this decision-making process. A new biological approach through multiple platelet-rich plasma injections is also gaining popularity lately (*76*). The goal of achieving optimally efficient and safe outcomes with biological therapy opens a novel niche for laboratory medicine in a comprehensive and multidisciplinary ATR management.

## Conclusions

Laboratory medicine in sport and exercise has significantly developed during the last decades with the awareness that physical activity contributes to improved health status, and is present in monitoring both professional and recreational athletes. Laboratory medicine extends to almost every aspect of sports, even to prevention of injuries but with less extent when the injury has occurred.

Training and competitions can modify concentrations of a variety of laboratory parameters, so the accurate laboratory data interpretation includes controlled and known preanalytical and analytical variables to prevent misleading interpretations. A corresponding “to-do” list in front of laboratory medicine specialists is extensive and challenging. Its core has four elements: continuous knowledge improvements about sport (patho)biology, resolving challenges brought by new analytical technologies, collaboration with other healthcare professionals involved in the field, and competencies to educate patients on minimizing the adverse effects of their sports and recreational activities on lab test results.

## Data Availability

No data was generated during this study, so data sharing statement is not applicable to this article.
